# Comparing Attention to Socially-Relevant Stimuli in Autism Spectrum Disorder and Developmental Coordination Disorder

**DOI:** 10.1007/s10802-017-0393-3

**Published:** 2018-01-09

**Authors:** Emma Sumner, Hayley C. Leonard, Elisabeth L. Hill

**Affiliations:** 1 Department of Psychology and Human Development, UCL Institute of Education, University College London, 25 Woburn Square, London, WC1H 0AA UK; 2 School of Psychology, University of Surrey, Guildford, UK; 3 Department of Psychology, Goldsmiths, University of London, London, UK

**Keywords:** Autism spectrum disorder, Developmental coordination disorder, Eye tracking, Face processing, Social, Attention

## Abstract

Difficulties with social interaction have been reported in both children with an autism spectrum disorder (ASD) and children with developmental coordination disorder (DCD), although these disorders have very different diagnostic characteristics. To date, assessment of social skills in a DCD population has been limited to paper-based assessment or parent report. The present study employed eye tracking methodology to examine how children attend to socially-relevant stimuli, comparing 28 children with DCD, 28 children with ASD and 26 typically-developing (TD) age-matched controls (aged 7–10). Eye movements were recorded while children viewed 30 images, half of which were classed as ‘Individual’ (one person in the scene, direct gaze) and the other half were ‘Social’ (more naturalistic scenes showing an interaction). Children with ASD spent significantly less time looking at the face/eye regions in the images than TD children, but children with DCD performed between the ASD and TD groups in this respect. Children with DCD demonstrated a reduced tendency to follow gaze, in comparison to the ASD group. Our findings confirm that social atypicalities are present in both ASD and to a lesser extent DCD, but follow a different pattern. Future research would benefit from considering the developmental nature of the observed findings and their implications for support.

The development of social skills, including socio-cognitive abilities and being able to establish and maintain friendships, plays a major role throughout development. Interacting with others requires interpreting a range of behaviours, both verbal and non-verbal. For example, the ability to interpret gaze can provide subtle clues as to what another person is thinking or attending to (Thorup et al. 2016), and developing selective attention to socially-relevant information, such as facial expressions, is crucial to understanding others and their intentions (Shepherd 2010). However, the task of processing these non-verbal cues is difficult; the information changes quickly and often, and thus may be more challenging than processing facial identity. Indeed, the complexity of face processing has been acknowledged in neuropsychological and neural models (Bruce and Young 1986; Haxby et al. 2000). Notably, processing this changeable information (i.e., expression, gaze) relies on attending to the relevant parts of the face at the right time, and a failure to do so is likely to result in problems in social interaction.

Difficulties with social interaction are not uncommon. In particular, individuals with autism spectrum disorder (ASD) are diagnosed based on marked impairments in social communication and interaction, along with restricted interests and repetitive behaviours (American Psychiatric Association [APA], 2013). In addition, although diagnosed on the basis of motor coordination difficulties (APA, 2013), research has indicated that children with developmental coordination disorder (DCD) also experience a range of social problems (Leonard 2016). The aim of the current study is to explore how children with ASD and children with DCD view socially-relevant information to understand whether the social problems evident in the two disorders rely on similar cognitive processing of social information. Increasing our understanding of the processes underlying similar behavioural outcomes in neurodevelopmental disorders is a vital step in improving diagnosis and treatment, allowing more focused interventions.

## Research on Social Attention and Face Processing in ASD

Research has highlighted difficulties with face recognition (see Chawarska et al. 2003) and problems with accurately processing emotions and gaze behaviour (Corbett et al. 2014; Dawson et al. 2005) as prominent characteristics of ASD. Further, during the last decade, the increased accessibility and accuracy of eye tracking technology has enabled more detailed exploration of how individuals with ASD view socially-relevant information (see Falck-Ytter et al. 2013; Guillon et al. 2014, for a review). Fixations and eye gaze patterns (i.e., scanpaths) provide a proxy for attention, as eye movements indicate overt orientation of visual attention. A number of studies have reported reduced attention to social stimuli. In particular, limited gaze to people and faces has been reported early in development, in toddlers and infants who are later diagnosed with ASD (Chawarska et al. 2013; Chawarska and Shic 2009; Sasson et al. 2011; von Hofsten et al. 2009; although see Elsabbagah et al. 2012). Reduced attention to another person’s face or eyes is believed to have repercussions for recognising subtle social cues, such as expressions and gaze (Falck-Ytter et al. 2013) and, therefore, could be argued to be an explanation for difficulties with social interaction in ASD.

However, inconsistencies exist in the adolescent and adult ASD literature on social attention and social processing (i.e., making sense of social cues, such as gaze). Some studies report a preference for fixating on non-social parts of a scene (i.e., the background and not the faces in the scene) when viewing social scenes in video clips (Klin et al. 2002; Speer et al. 2007), photographs of human faces (Dalton et al. 2005; Riby and Hancock 2009; Sasson et al. 2007) and naturalistic photographs of social scenes (Riby and Hancock 2008). There have been reports of reduced time looking at the eyes (Dalton et al. 2005; Riby and Hancock 2008, 2009; Sasson et al. 2007) and Freeth et al. (2010, 2011) found individuals with ASD were slower to initially fixate the face in a photograph. Slower fixations to the face region were considered to provide some indication of a reduced interest in social aspects of the scene. In addition, difficulties with processing social cues have been reported, as it has been found that adolescents with ASD rarely spontaneously follow an actor’s gaze in a photograph (Riby et al. 2013; Ristic et al. 2005) and preschoolers with ASD did not respond to gaze cues to an object shown in video clips (Vivanti et al. 2014). Others, however, report no avoidance of the face region, nor problems with gaze following in high-functioning young adults with ASD (Fletcher-Watson et al. 2009; Freeth et al. 2010, 2011). A possible explanation for mixed findings may be due to differing methodologies, the heterogeneous nature of an ASD population, or possibly the development of compensatory strategies in some individuals, in response to social experiences in early life. Of note, very little is known about the viewing patterns of primary-aged children with ASD, and knowledge of how this age group process social information would contribute to the current body of work seeking to understand the development of such skills.

## Research on Social Skills and Cognition in DCD

A number of studies have flagged poor social skills in children with DCD. Although primarily a motor disorder, research indicates that children with DCD experience additional problems with peer relations (Chen et al. 2009; Dewey et al. 2002; Poulsen et al. 2007; Smyth and Anderson 2000; Wagner et al. 2012) and difficulty processing emotions from the face (Cummins et al. 2005). A recent study by Sumner and colleagues (Sumner et al. 2016b) directly comparing children with ASD and those with DCD highlighted that the two groups were comparable on paper- and laptop-based face processing tasks in their ability to identify facial expressions, speech sounds, and gaze; with both groups performing worse than a control group. In addition, parents reported lower social functioning (i.e., building friendships, engaging in play activities) in the DCD and ASD groups compared to typically-developing children, with ASD children the most compromised. The authors were able to demonstrate that motor ability predicted social functioning in these two clinical groups, providing a possible explanation for why social difficulties are found in DCD. This is in line with Campos et al. (2000), who have argued that the development of early gross motor skill (sitting, crawling, walking) provides increasing opportunities for infants to interact with the world and others. As infants become independent explorers, through crawling and walking, they develop skills for social referencing and interaction (Clearfield et al. 2008). Considering the development of children with DCD, it has been shown that these children are significantly delayed in achieving early gross motor milestones (Sumner et al. 2016b) and, therefore, they may well be missing out on vital opportunities to develop socially. Thus, although social interaction difficulties may appear similar in DCD and ASD, they may differ in the causal pathways and subsequent developmental trajectory of social skill development.

The social abilities of children with DCD should be explored further. To date, no study has used eye tracking methodology to examine how children with DCD attend to socially-relevant stimuli. Although similarities have been identified between children with DCD and ASD in terms of the number of correct responses they make when identifying socially-relevant information, such as expressions and gaze, (Sumner et al. 2016b), it is possible that this behavioural similarity relies on different attention and processing in the two groups. Given that social difficulties may persist through to adolescence and adulthood in DCD, there is a need to better understand the social problems experienced by this population, which should increase recognition and inform appropriate intervention.

## The Present Study

Direct comparisons of neurodevelopmental disorders can help to ascertain whether behavioural and cognitive phenotypes are specific or general to a disorder. The overall aim of the present study was to employ eye tracking methodology to examine how children with ASD, those with DCD, and typically-developing (TD) children view socially-relevant information. In doing so, we contribute to the existing eye tracking literature on ASD, which is currently lacking with regards to knowledge of primary-aged children. We also further our understanding of DCD by using this methodology, which has not yet been used to analyse social behaviour within this population.

In the present study, spontaneous gaze was recorded and later analysed while participants viewed images classified as ‘Individual’ (one person in the scene) and ‘Social’ (two or more people, depicting an interaction). For the ‘Individual’ images, a standard approach from previous research was used, in which faces are presented in a frontal view, with direct gaze towards the participants (e.g., Pelphrey et al. 2005; Speer et al. 2007). This allowed a direct comparison of individuals in the current ASD group to those in previous research in terms of the relative time spent looking at the eyes or face compared to other parts of the scene. It also allowed the exploration of the allocation of attention to these regions of interest in the DCD group. However, this is not the most naturalistic task, and therefore the social scenes were included to assess attention to socially-relevant elements of more ecologically-valid stimuli and address the limitations of these more controlled images. Social scenes used in previous studies (e.g. Riby and Hancock 2008; Riby et al. 2013) have used naturalistic photographs of people during an interaction, in which gaze is often averted from the participant and appears more naturalistic between people presented in the scene. The current study adopted this approach for the ‘Social’ stimuli, presenting naturalistic interactions between children. In addition to identifying the regions of interest in social scenes, these stimuli also allowed for consideration of gaze following behaviour. The analyses therefore aimed to address a number of research questions:

1. Do children with ASD, children with DCD, and TD children differ in the amount of time they fixate on the face and eye regions in a social scene?
2. Does symptom severity (i.e., ASD symptomology, and motor skill in DCD) relate to the amount of time children fixate the face?
3. Do children with ASD and those with DCD take longer to first fixate the face region, in comparison to TD children?
4. Do the three groups show evidence of spontaneously following a person’s gaze?

Based on previous research, a number of hypotheses were proposed. We predicted that children with ASD would fixate for less time on faces, and the eye region, and be slower to first fixate the face region than their TD counterparts. It was also hypothesized that children with ASD would be poorer at following gaze to an object than TD children. Children with DCD were expected to demonstrate a similar profile to children with ASD, although to a lesser extent, and thus perform below that of the TD group.

## Method

### Participants

Participants aged 7–10 years formed three groups: children with ASD, children with DCD, and TD children. Groups were matched on chronological age (*p* = 0.15). Importantly, children in the two clinical groups (ASD and DCD) had an existing diagnosis from relevant clinicians external to the research team. Diagnoses were corroborated - and ruled out in the TD group - using various background measures and the following inclusion/exclusion criteria applied (see Table 1 for group characteristics):

1. All participants had a Full Scale IQ (FSIQ) ≥80 on the Wechsler Intelligence Scales for Children (WISC-IV; Wechsler 2003).
2. Parents completed the lifetime version of the Social Communication Questionnaire (SCQ, Rutter et al. 2003). The SCQ contains 40 questions, half of which focus on the behaviour of the child at their present age and the other half of questions relate to their child’s behaviour during the period of time between their 4th and 5th birthday. A score of 15 and above is suggestive of ASD symptomology. All children in the ASD group were required to score ≥ 15, while all TD children had to score below the cut-off 15 (i.e., ruling out ASD characteristics in this group). In fact, all TD children scored below 9 on the SCQ (note: 72% of the TD group scored <5). The SCQ was an exploratory measure for the DCD group and, therefore, no cut-off was applied.
3. Motor competency was assessed using the Movement Assessment Battery for Children, second edition (MABC-2; Henderson et al. 2007), a standardised assessment of fine and gross motor skill. Overall test performance was converted to a percentile rank (UK norms). As all children in the DCD group had an existing diagnosis, the MABC-2 inclusion criterion for this group was a score ≤ 16th percentile. Children in the TD group scored ≥25th percentile. The MABC-2 was an exploratory measure for the ASD group and thus no exclusion criteria were applied.
4. Prior to testing, parents completed a screening questionnaire. Parents of children in the ASD and DCD groups reported no additional diagnoses, such as ADHD, ASD, or dyslexia, no form of visual or neurological impairment, nor a general medical condition; parents of children in the TD groups did not identify diagnoses of any kind.

**Table 1:** Participant characteristics

Characteristics	TD (*n* = 25)	ASD (*n* = 28)	DCD (*n* = 28)	*F(df)*	*p*	*n* ^2^_*p*_	Post hoc
Gender (m;f)	22;3	24;4	21;7	–	–	–	–
Age (in years)							
Mean (SD)	9.10 (1.07)	8.58 (1.18)	8.53 (1.16)	1.97	0.15	0.05	All *ns*
Range	7.70–10.74	7.01–10.91	7.04–10.99	(2, 78)			
FSIQ standard score							
Mean (SD)	110.64 (10.07)	101.32 (14.32)	95.93 (12.47)	9.30	0.001	0.19	(DCD = ASD) < TD
Range	89–127	80–136	80–126	(2, 78)			
MABC2%ile							
Mean (SD)	63.20 (22.20)	31.87 (32.42)	3.23 (4.87)	47.03†	0.001	0.58	DCD < ASD < TD
Range	25–98	0.01–95	0.01–16				
SCQ							
Mean (SD)	2.84 (2.67)	22.82 (6.13) ^a^	9.85 (6.43) ^a^	92.05	0.001	0.71	TD < DCD < ASD
Range	0–9	15–38	1–27	(2, 76)			

FSIQ = Full Scale IQ from the WISC, *M* = 100, *SD* = 15. MABC-2 = Movement Assessment Battery for Children, percentile scores; SCQ = Social Communication Questionnaire. ^a^1 missing data point because parents did not return the questionnaire. *ns* = non-significant. †Nonparametric analyses conducted due to unequal variances (Kruskal-Wallis *H* and post hoc Mann-Whitney reported)

Thirty-one children with ASD were recruited through specialist schools and special educational needs units attached to mainstream schools in South London, and also by advertisements through a charitable organisation, the National Autistic Society. One child was excluded due to a low FSIQ, two children were excluded because they had difficulty completing the eye tracker calibration procedure (following four attempts). The final sample comprised 28 children (24 male) with ASD. As well as considering information from the SCQ, children in this group scored ≥7 on Module 3 of the Autism Diagnostic Observation Schedule (ADOS-2; Lord et al. 2012), demonstrating a group mean score of 8.67 (standard deviation, 1.11). ADOS data were not collected for 3 children in the sample because they had undergone their diagnostic assessment, which required completing the ADOS, close to the time of the study. Fourteen children with ASD (50%) had moderate to significant motor difficulties (≤16th percentile) as assessed by the MABC-2.

The DCD group initially comprised 34 children, recruited via an advertisement placed with a charitable foundation, the Dyspraxia Foundation, and primary schools in South London. One child was excluded due to a FSIQ below cut-off, and a further 5 participants were removed from the sample due to difficulties with eye tracking calibration requirements (again following four attempts to calibrate). Therefore, the final sample comprised 28 children (21 male) that met the DSM-5 criteria (APA, 2013) for DCD: motor ability below the level expected given the child’s age and measured-IQ, motor difficulties were not explained by medical or neurological condition and were present in the early developmental period. Of note, 5 children with DCD (18%) scored above the SCQ cut-off.^1^

Thirty-one TD children were recruited from mainstream primary schools in the South London area. Adhering to the inclusion/exclusion criteria, six children were excluded from further study due to scoring below the motor difficulty cut-off on the MABC-2. The final TD sample group thus consisted of 25 children (22 male).

### Procedure

Ethical approval was obtained from Goldsmiths, University of London. All schools and parents of the participating children were provided with detailed information relating to the study aims, planned tasks and the procedures relating to maintaining anonymity for each participant. Informed consent was then obtained by asking head teachers of the schools and the individual parents to provide written consent confirming that they were happy for the children to take part in the study. Further, all children gave verbal assent after the tasks were explained in full.

### Materials and Apparatus

The stimuli consisted of 30 colour images. They were stock images selected and purchased for the purpose of the experiment through Fotolia. Each image was landscape orientation and was presented at 800 × 600 pixels. The stimuli depicted a range of social activities and included children of a similar age to the participants in the study.

**Fig. 1:**
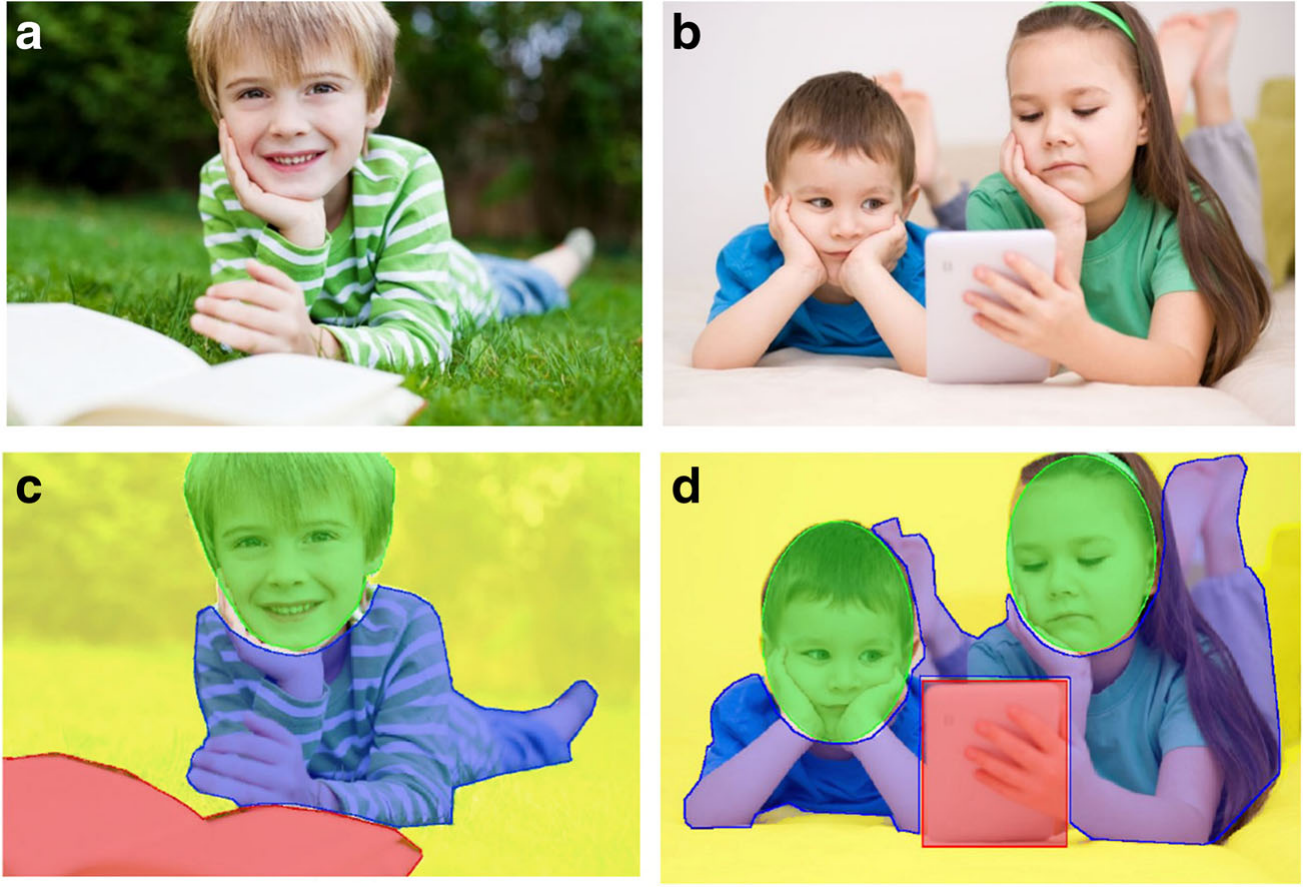
Stimuli examples of (**a**) an ‘Individual’ image (a: © contrastwerkstatt/Fotolia), and (**b**) a ‘Social’ image. (b: ©julaszka/Fotolia). AOI examples (**c** and **d**) are also shown. Yellow shading indicates the background of the image, red represents the object AOI, the green is the face AOI, and blue represents the body AOI

Stimuli were randomly divided into two sets of images: 15 images showing an ‘Individual’ character with their attention directed towards the viewer; and 15 images showing ‘Social’ interactions between two or more characters (maximum, 4, following a similar procedure to Riby and Hancock 2008). In the latter, the characters’ attention (gaze) was directed to others or objects in the scene. All images contained either one character (child) presented close to an everyday object (‘Individual’), or two or more characters interacting with the object (i.e., with gaze directed towards it; ‘Social’). The object was considered a non-social aspect of the scene, along with the background of the scene, while the head and body of the character(s) were considered to be social elements. Example ‘Individual’ scenes included a child sitting with a book open but looking directly at the camera (see Fig. 1a), a child on a scooter, etc.; and ‘Social’ scenes included children playing on a tablet (see Fig. 1b), throwing a ball to each other, reading together. Images were presented in a random order and divided into 2 blocks of 15 (mixed Individual/Social).

Eye movements were recorded using the Eyelink 1000 (SR-research; 1000 Hz sampling rate), and children viewed the stimuli presented on a computer monitor from a viewing distance of approximately 70 cm (1024 × 786 screen resolution). Each image was presented for 3000 ms. The stimuli subtended a visual angle of approximately 29 × 18°. At the beginning of the session, children completed a 9-point calibration, requiring fixations to be made within 1° of each fixation point, followed by a validation phase. This procedure was also repeated after the break that followed the first block of images. The camera was set up in the desktop mount and was non-invasive. A combined forehead/chin rest was used to keep the head stable and the eye movements within range. All children were given the option to not use this rest, but all opted to do so. A height-adjustable chair was used and the forehead/chin rest could also be adjusted for height and size to accommodate the participant. The experiment was implemented using Experiment Builder and analysed using Data Viewer (both SR Research software). Data Viewer automatically identifies fixations and saccades using predetermined criteria. Saccades are operationalised by velocity/acceleration criteria (30° per second; acceleration > 8000° per second squared); fixations are determined as when velocity drops back below 30° per second.

### Eye Tracking Data Analyses

#### Areas of Interest (AOIs)

Data Viewer (SR-research) enabled the construction of AOIs over the viewed images. The first step of analysis identified four AOIs: the background in the image, the object, the face, and the body. Figure 1c and d provides an example of how AOIs were drawn in the first instance. This was then followed up to consider the eye and mouth regions/AOIs. Analyses were conducted to investigate fixations to specific AOIs. Following a similar procedure to Riby and Hancock (2008), fixations to the ‘background’ were calculated by drawing an AOI around the outline of the image and calculating all fixations to the image minus those directed to the other AOIs (face, body, object). Therefore, ‘background’ does not include the white border of the screen surrounding the image (i.e., the image did not fill the whole screen). A recent meta-analysis identified that percentage of time spent in specific regions is the most widely and uniformly reported measure in eye tracking studies that focus on ASD population (Chita-Tegmark 2016). Therefore, overall time spent fixating the AOIs was calculated as a percentage of the time that the image was shown on screen.

#### Time to Fixate the Face

This was calculated from the start time at which the image was presented on the screen in relation to when a fixation was first made to any part of the face AOI (in seconds). First fixations were recorded as those that occurred after the first saccade from the stimulus onset. This prevented scoring fixations that occurred in the face region simply because the eye position was there when the image was first shown.

#### Gaze Following

The Social images contained 2 or more children and their gaze was naturally directed either to another child or the object in the scene. For ease of analysis, tendency to follow gaze was investigated only during the images that showed all children in the scene directing their gaze to the same object. Six images met this criteria (the remaining images had mixed content - where children in the scene were looking at different things, either the object or another child). Figure 1b is an example of two children both looking at a tablet device. Other examples include children reading a book together, passing a ball, and completing a puzzle. Gaze following was confirmed if children fixated on the eye AOI of the children in the photographs and then executed a saccade immediately to the object AOI. Additional analyses were conducted on scan paths where children fixated on the head AOI followed by the object AOI. Number of valid gaze shifts were recorded for each participant, as well as the time spent fixating the object.

### Statistical Analyses

Tests of normality and homogeneity were checked prior to statistical test selection. Parametric tests were conducted unless otherwise indicated. To answer research questions 1 and 3, repeated measures ANOVAs were conducted to investigate group differences and to compare Individual and Social images. Significant main effects and interactions were analysed further using post-hoc comparisons and simple main effects. Bonferroni-corrected values are indicated in the relevant sections of the results. To answer research question 2, bivariate correlations were conducted. Sensitivity analyses revealed that *N = 81* would be sufficient to detect a small to medium effect size for both types of analyses (Faul et al. 2007).

Table 1 revealed that children in the TD group had a significantly higher FSIQ than children in the ASD and DCD groups, while the clinical groups were comparable. In line with existing ASD eye tracking studies (Fletcher-Watson et al. 2009), correlations between FSIQ and the dependent variables were considered. However, no significant correlations were found between FSIQ and the eye tracking measures (i.e., total fixation time, time to fixate an AOI, etc). Similarly, correlations between age and gender and the eye tracking measures were considered, but revealed non-significant results (*p*s > 0.12). Therefore, FSIQ, age, and gender were not included as covariates in the subsequent analyses.

## Results

Following the same criteria applied by Fletcher-Watson et al. (2009), trials with less than 500 ms of eye tracking data recorded were excluded from the analysis. Using this criterion, only 1.3% of the trials were removed and these were roughly split across the three groups. A univariate ANOVA revealed that participants in each group were engaged with the task (as measured by total fixation durations per image) for comparable amounts of time in the Individual (*F*(2, 78) = 1.68, *p* = 0.10, *n*^2^_*p*_ = 0.04: TD group *M* = 2.56 s per image, SD = 1.97; ASD group *M* = 2.34 s per image, SD = 0.46; DCD group *M* = 2.60s per image, SD = 0.36) and Social images (*F*(2, 78) = 0.82, *p* = 0.44, *n*^2^_*p*_ = 0.02: TD group *M* = 2.71 s per image, SD = 0.23; ASD group *M* = 2.33 s per image, SD = 0.43; DCD group *M* = 2.63 s per image, SD = 0.27). When participants were not engaged in the task (i.e., fixating on the screen), they were making saccades, blinking, or looking away from the screen.

A 2 × 3 (Image Type x Group) ANOVA was conducted on the number of saccades made per trial (Individual images: TD group *M* = 11.16, SD = 1.36, ASD group, *M* = 10.45, SD = 1.87, DCD group, *M* = 9.97, SD = 1.73; Social images: TD group *M* = 12.25, SD = 1.38, ASD group, *M* = 11.37, SD = 2.05, DCD group, *M* = 10.99, SD = 1.72). Significantly more eye movements (saccades) were made in the Social images, *F*(1, 78) = 144.56, *p* < 0.001, *n*^2^_*p*_ = 0.65, presumably because the images contained more information (characters) to look at. A significant effect of group was seen, *F*(2, 78) = 3.77, *p* = 0.03, *n*^2^_*p*_ = 0.08, with post hoc analyses revealing no differences for the TD vs ASD and DCD vs ASD comparisons (*p*s > 0.24), but children with DCD made significantly fewer saccades than the TD group (*p* = 0.02). No interaction was found, *F*(2, 78) = 0.36, *p* = 0.69, *n*^2^_*p*_ = 0.01. Given that the number of saccades made was different only for the DCD and TD comparison, the correlation between the number of saccades and the variables tested in subsequent analyses was taken into consideration. However, no significant correlations were found between number of saccades per trial and any of the experimental eye tracking measures (viewing time to the AOIs, time to first fixate the face). For this reason, the number of saccades made was not included as a covariate in the subsequent analyses.

### Viewing Times to Areas of Interest

**Fig. 2:**
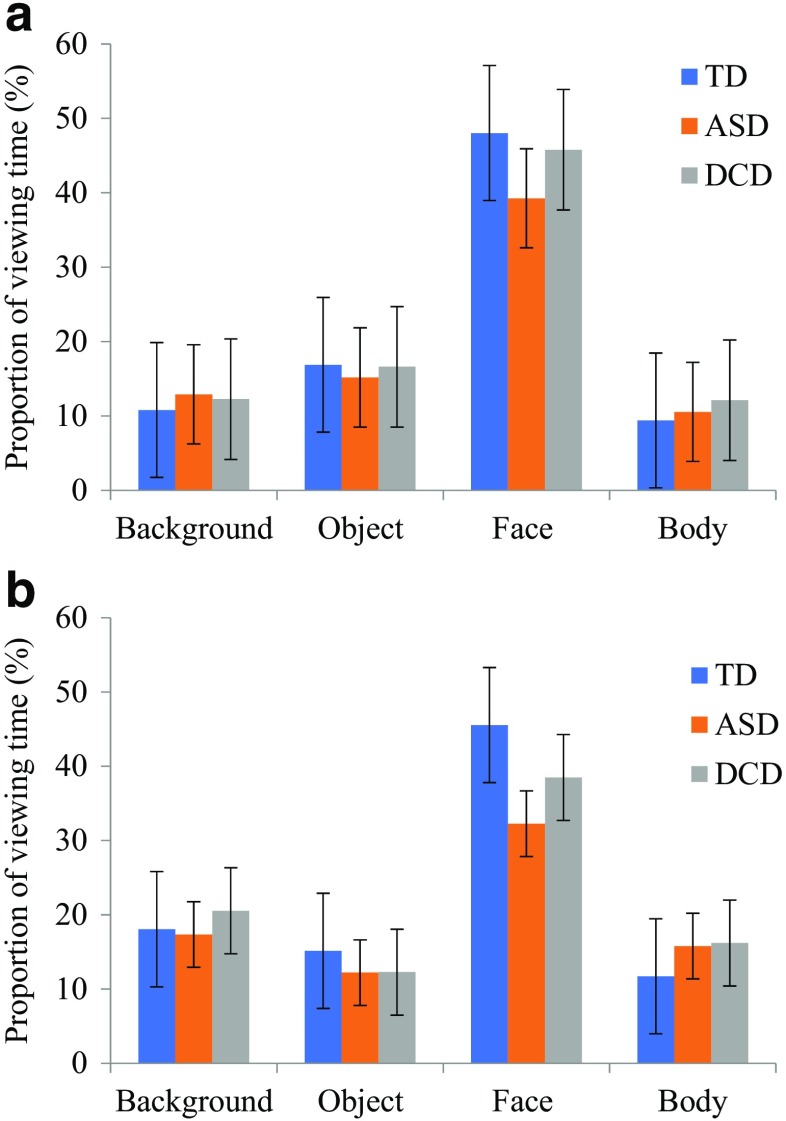
Gaze to areas of interest for (**a**) Individual and (**b**) Social across the three groups. The proportions do not total 100% because of time spent looking at areas outside of the interest areas

Figure 2 illustrates how visual attention was distributed across the Individual and Social scenes, considering percentage of total viewing time per trial to the following AOIs: background, object, face, body.

A 2 × 4 × 3 (Image Type x AOI x Group) ANOVA was conducted. Significance levels were Bonferroni-corrected to *p* = 0.01. There was no main effect of Image Type, *F*(1, 78) = 5.70, *p* = 0.02, *n*^2^_*p*_ = 0.07, but there was a main effect of AOI, *F*(3, 234) = 272.53, *p* < 0.001, *n*^2^_*p*_ = 0.78, revealing a larger proportion of time spent fixating the face region in comparison to the other AOIs (*p* < 0.001). A significant interaction between Image Type and AOI, *F*(2, 234) = 58.72, *p* < 0.001, *n*^2^_*p*_ = 0.43, was found, which showed longer viewing time to the face in the Individual images than the Social images, and more time spent on the background in the Social images than in the Individual images.

There was a main effect of Group, *F*(2, 78) = 7.49, *p* = 0.001, *n*^2^_*p*_ = 0.16, and a significant interaction between AOI and Group, *F*(6, 234) = 4.49, *p* < 0.001, *n*^2^_*p*_ = 0.10, but not for Image Type and Group (*p* = 0.02). Children with ASD did not differ from the TD group in viewing time to the background, object, or body AOIs in both image types (all *p*s > 0.14), but they did fixate on the face significantly less than TD children in the Individual, *t*(51) = 3.04, *p* = 0.003, *d* = 0.84, and Social images, *t*(51) = 4.68, *p* < 0.001, *d* = 1.28. Compared to TD children, children with DCD were comparable in their viewing times to all AOIs (all *p*s > 0.03) in the Individual images and all in the Social images, except that children with DCD spent significantly longer looking at the body AOI than TD children *t*(51) = 2.88, *p* = 0.005, *d* = 0.80. The DCD and ASD groups did not differ in attention to any of the AOIs (all *p*s > 0.07).

**Fig. 3:**
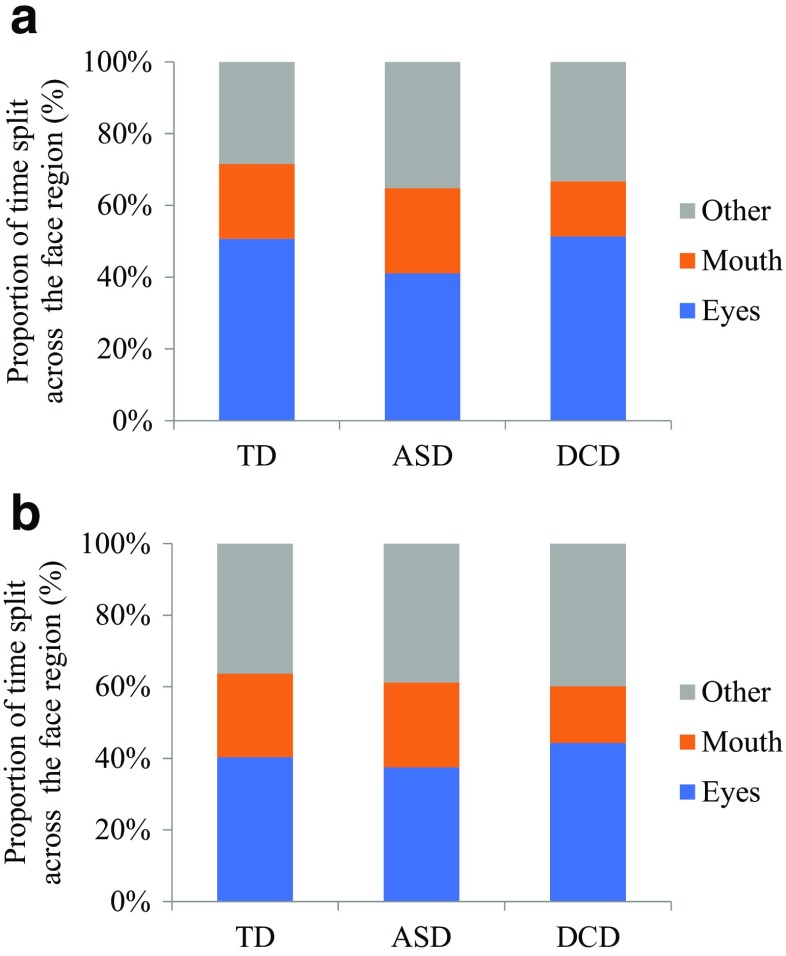
Gaze time directed to the eyes, mouth, and other AOI in the face region, as a proportion of time spent fixating the face as a whole, for (**a**) Individual and (**b**) Social images across the three groups

The next stage of analysis considered precisely where participants were looking when fixating on the face (see Fig. Fig. 3). Statistical comparisons were made only between the eyes and mouth region (not the ‘other’/remaining part of the face). A 2 × 2 × 3 (Image Type x AOI x Group) ANOVA was conducted. Significance levels were Bonferroni-corrected to *p* = 0.01. There was a main effect of Image Type, *F*(1, 78) = 54.33, *p* < 0.001, *n*^2^_*p*_ = 0.41, such that a greater proportion of viewing time was spent in the identified AOIs in the Individual images across all groups. There was also a main effect of AOI, *F*(1, 78) = 48.69, *p* < 0.001, *n*^2^_*p*_ = 0.38, revealing a larger proportion of time spent fixating the eye region in comparison to the mouth; and a significant interaction between Image Type and AOI, *F*(1, 78) = 14.67, *p* < 0.001, *n*^2^_*p*_ = 0.16, which showed a greater difference between fixating the eyes and mouth in the Individual images. There was a main effect of Group, *F*(2, 78) = 5.89, *p* = 0.004, *n*^2^_*p*_ = 0.13, revealing children with ASD spent significantly less time fixating on the eyes (*p* < 0.01) than the TD group; but no significant interactions involving Group (*p*s > 0.08).

### Correlations with Symptom Severity

Time spent fixating the face region was combined for both the Individual and Social performance. A significant negative correlation was found for the SCQ score (all groups combined for a full range of scores) and overall time spent fixating the face (*r* = −0.32, *p* = 0.006). Higher scores on the SCQ indicate more ASD-like symptomology, thus those recognised by parents as having more social difficulties spent less time fixating the face region. However, when correlations were split by group, no significant correlations remained for the SCQ and overall time spent fixating the face (*p*s > 0.11). The MABC-2 total test score did not correlate with the overall time spent fixating the face region (*p* = 0.13), suggesting no relationship between motor ability and a preference to fixate the face. Of note, the SCQ and MABC-2 performance were not significantly correlated with the subsequent eye tracking measures: time to fixate the face, or gaze following.^2^

### Time Taken to Fixate the Face

Table 2 reports the time taken to first fixate the face region in the images. The correlation between the number of saccades made during the trials (reported at the beginning of the results) and the time taken to fixate the face region was considered but revealed a non-significant relationship (*r*s < 0.42 and *p*s > 0.93). Therefore, a link between more saccades and longer times to fixate the face was not found.

**Table 2:** Mean time taken, in seconds, to fixate the face: all groups

Time to fixate	TD (*n* = 25)	ASD (*n* = 28)	DCD (*n* = 28)
Individual			
Mean (SD)	0.53 (28)	0.59 (0.14)	0.75 (0.40)
Range	0.29–1.31	0.37–0.93	0.40–1.89
Social			
Mean (SD)	1.17 (0.55)	1.13 (0.18)	1.15 (0.48)
Range	0.55–1.92	0.78–1.47	0.46–2.07

A 2 × 3 (Image Type x Group) ANOVA revealed no effect of group membership, *F*(2, 78) = 1.41, *p* = 0.25, *n*^2^_*p*_ = 0.04, although a signficiant effect of image type was found, *F*(1, 78) = 125.47, *p* < 0.001, *n*^2^_*p*_ = 0.62. All groups fixated the face sooner in the Individual images. Further, a non-significant interaction, *F*(2, 78) = 1.83, *p* = 0.17, *n*^2^_*p*_ = 0.05, demonstrated that this effect was similar across groups. Nevertheless, it was noteworthy that, although not statistically significant, children with DCD took longer to fixate the face than the TD and ASD groups in the Individual images.

### Gaze Following

**Fig. 4:**
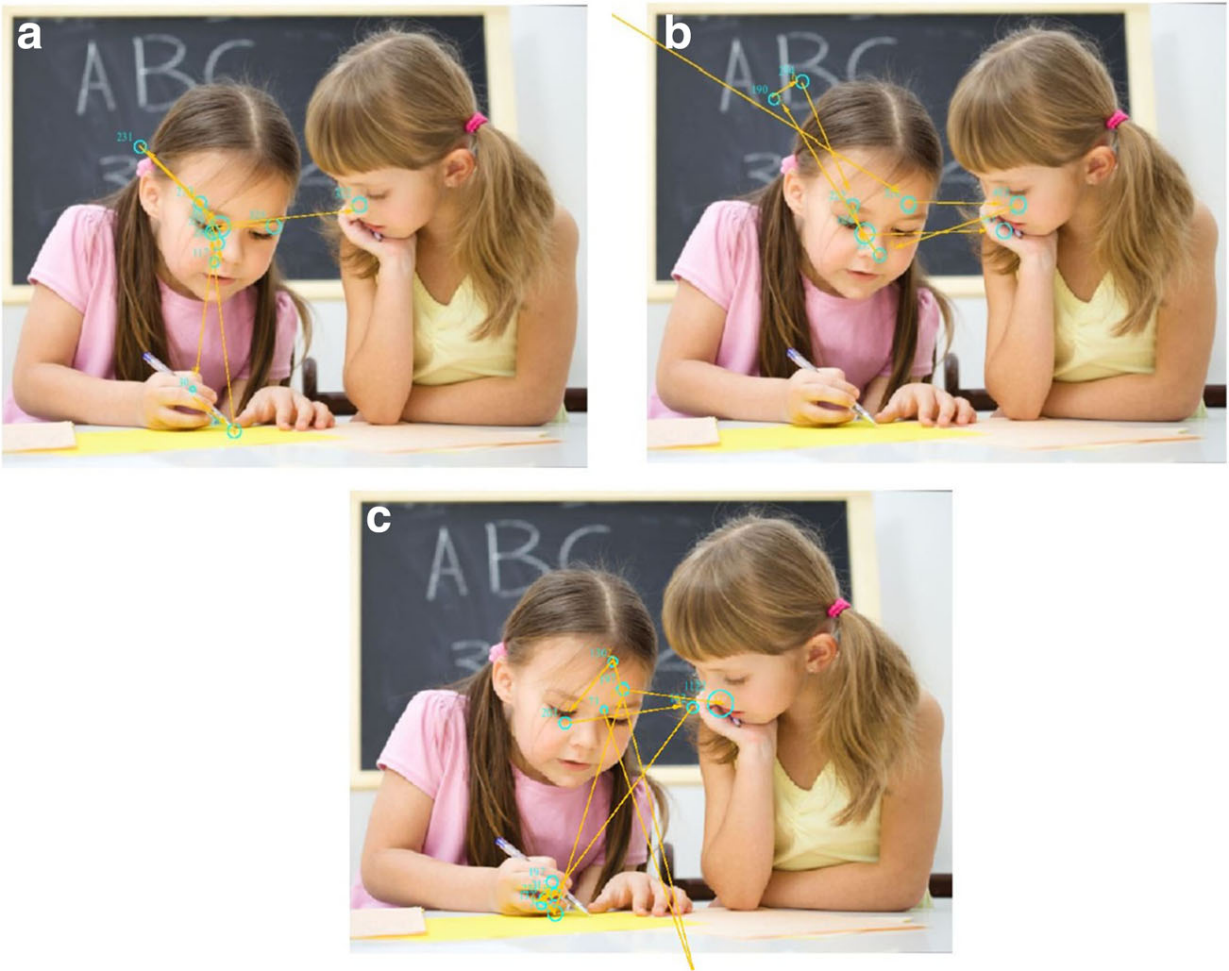
Examples of gaze scan paths by (**a**) TD child, (**b**) child with DCD and (**c**) child with ASD. Photograph © Serhiy Kobyakov/Fotolia

Example scanpaths are shown in Fig. 4. As a reminder, this analysis was focused on 6 Social images. Of note, 1 TD child (3.8% of the group) did not make any valid gaze shifts (eye AOI to object AOI) in any of the six images, neither did 6 children with ASD (21%) and 8 children with DCD (29%). Of those children who did follow gaze at least once, the mean number of valid gaze shifts from the eye to object AOI for the TD group was *M* = 3.00, SD, 1.93 (range: 1–6), in the ASD group, *M* = 2.26, SD, 1.37 (range: 1–6) and in the DCD group, *M* = 2.56, SD, 1.15 (range: 1–5). No significant group difference was found for the mean number of eye to object gaze shifts, *F*(2, 67) = 1.06, *p* = 0.35, *n*^2^_*p*_ = 0.04. Time spent fixating the object was analysed as an indicator of interest in the part of the scene to which the actors were directing attention, and also revealed no significant group differences (*F*(2, 75) = 259, *p* = 0.06. *n*^2^_*p*_ = 0.06): TD, *M* = 0.60s per image, SD = 0.27; ASD, *M* = 0.46 s per image, SD = 0.26; DCD, *M* = 0.44 s per image, SD = 0.25).

Given that the direction/angle of the head can also provide cues as to where attention is directed, we extended this analysis to look at gaze shifts from the face to object. Here we found that all TD children made valid gaze shifts from the head AOI to the object AOI, while 2 children with ASD (7%) still did not and, interestingly, 5 children with DCD (18%) failed to do so: suggesting that a proportion of children were not receptive to following others’ visual attention. When the analysis was not constrained to the eye region, a higher number of gaze shifts were found in all groups (TD, *M* = 4.84, SD, 2.05 (range: 1–8); ASD, *M* = 3.35, SD, 1.76 (range: 1–6); DCD, *M* = 3.54, SD, 1.77 (range: 1–8). Here significant group differences were found, *F*(2, 78) = 6.70, *p* = 0.002, *n*^2^_*p*_ = 0.12 and post hoc analyses revealed that children with DCD (*p* = 0.005) and children with ASD (*p* = 0.007) made significantly fewer gaze shifts from the head to object AOI than the TD group. However, the DCD and ASD groups made a comparable number of gaze shifts (*p* = 0.99).

## Discussion

The main aim of the present study was to examine how children with ASD, DCD and TD children view socially-relevant information, and to determine whether groups differed in this respect. Individual images showing direct gaze were used alongside scenes that were more ecologically valid in showing a natural interaction among two or more children. Confirming our initial predictions, and in support of exisiting findings on younger and older ASD populations (Klin et al. 2002; Riby and Hancock 2008, 2009; Sasson et al. 2007; von Hofsten et al. 2009), primary-aged children with ASD were found to fixate less on the face and eye regions than TD children. This may, in part, contribute to the difficulties these children often experience with recognising faces and processing facial expressions and gaze (Corbett et al. 2014; Dawson et al. 2005; Sumner et al. 2016b). However, in comparison to TD children, children with ASD were found to take a similar amount of time to first make a fixation to the face region, conflicting with exisiting data on adolescents with ASD (Freeth et al. 2010, 2011) and suggesting that children with ASD do search a scene for social information to some extent. Nevertheless, a number of children with ASD (21%) did not spontaneously follow the gaze (from the eye AOI to object) of the children in the images. Thorup et al. (2016) found that infants at-risk of ASD were less likely to follow gaze when information was only provided from an adult’s eye and instead they were more successful in doing so when a head shift was made alongside gaze. In the present study, the eyes/head in the images were always in the same direction, but we found that the percentage of children with ASD who did not demonstrate a gaze shift to the object decreased to 7% when looking at saccades made from anywhere in the head AOI to the object, indicating that some children may interpret clues from the angle of the head rather than the eyes.

Using eye tracking methodology to better understand social behaviour in a DCD population was a novel approach, and comparisons to ASD and TD children enabled the identification of some atypicalities. In fact, this study provides the first experimental account of how children with DCD process socially-relevant information. While the pattern of results were not as exaggerated for children with DCD as they were for children with ASD (they performed in between the TD and ASD groups on time fixating the head and eye regions), some interesting patterns of behaviour did emerge. Although not significantly different, overall children with DCD were slower than the ASD and TD groups to first fixate the face in the viewed images. Moreover, children with DCD were the most impaired out of the three groups in the examination of tendency to follow gaze. Nearly a third of the DCD group did not follow the direction of gaze from the eyes to the object on even one occasion. This decreased to 18% when the head-to-object analysis was conducted, indicating that a significant number of children with DCD did not utilise gaze cues when passively viewing the social interactions. It is interesting that although children with DCD, on average, spent longer looking at the eye region than those with ASD, fewer children with DCD interpreted these gaze cues. Gaze can reveal a person’s intentions and research has shown that other people’s eyes naturally guide our own visual attention (Macdonald and Tatler 2013). A lack of awareness in this respect may indicate poor understanding of social conventions and would understandably have repercussions for social interaction. Thus, these exploratory findings of unusual gaze behaviour potentially support the notion of social atypicalities in a DCD population (Cummins et al. 2005; Sumner et al. 2016b), and this work could be usefully extended with more specific gaze following tasks, discussed below.

In addition to the group comparisons discussed above, the current study also assessed the influence of broader social abilities on how children process socially-relevant information across the three groups. As expected, a higher number of atypical social characteristics, as measured by the parent report SCQ, were shown to correlate with fewer fixations made to the face region; although this correlation is likely driven by group membership, given that when considered by individual group the correlation diminished. The direction of a possible relationship, however, remains unclear; thus, reduced attention to faces may influence how children act socially or social experiences may influence how children with ASD and DCD attend to faces. Future research might further explore this relationship with more experimental measures of social skills, rather than parent reports, in relation to eye-tracking performance, over development, which will allow further interpretations of cause and effect in these relationships.

Unexpectedly, when considering the earlier argument that motor skills enable opportunities for social interaction (Campos et al. 2000; Clearfield 2011), poorer motor skill did not correlate with fixations to the face. The interaction between motor and social development may be better studied in early development, which can be difficult in a DCD population when diagnoses are often made from the age of 5 (Blank et al. 2011). Indeed, children with DCD are significantly delayed in achieving early gross motor milestones (Sumner et al. 2016b), and may therefore demonstrate clearer social differences earlier in life than was studied here. The fact that children with DCD in the current study did not differ significantly from the typically-developing group but performed at a slightly lower level overall across eye-tracking measures may suggest that the close relationship between motor and social difficulties in this group is mediated over time. The social difficulties reported in DCD at school age (e.g., Chen et al. 2009; Dewey et al. 2002) may thus be a consequence of poor motor skill and its effect on interaction, rather than the primary difficulty in social skills seen in ASD. Future research would benefit from exploring whether social difficulties (including difficulties following gaze cues) are present from an early age or emerge as a possible repercussion of motor problems in children with DCD, but also in an ASD population where motor difficulties are increasingly reported (Landa et al. 2013).

The present study was careful to select the sample groups so that co-occurring diagnoses were not present and, therefore, social atypicalities found in the DCD group cannot be attributed to these children having a dual diagnosis. However some limitations related to the methodology can be raised. One potential problem might be that the Individual and Social images differed in not only the the number of people in the image, but also in the gaze direction of those presented (i.e., direct gaze in the Individual images, and averted gaze in the Social images). Direct gaze has been shown to influence ASD performance, as atypical activation in theory-of-mind networks in adults with ASD has been shown when viewing direct gaze (Hamilton 2015; von dem Hagen et al. 2014) and increased physiological responses have been noted which may interfere with face processing (Joseph et al. 2008; Kylliainen and Hietanen 2006). However, this argument does not hold true in the present study as all groups made a higher proportion of fixations to the face (and eyes) region in the Individual images where the gaze was directed to the observer, than in the Social images where gaze was directed between the characters/objects in the images. In fact, for all groups, more saccades were made and participants were slower to first fixate the face region and directed fewer fixations to the face region in the Social images, suggesting that the content of the scene influences eye movements. Comparing highly-controlled direct-gaze images and more naturalistic social scenes within one study was important because it could help to explain the mixed findings from previous studies. Future research could take this further by comparing a range of different stimuli (e.g., individual and social images with both averted and direct gaze) across groups or consider whether the present findings translate to real-life scenarios. We live in a multi-sensory environment and it may be expected that these findings would be more pronounced in real life, where more distractions may occur and the information provided by the face changes rapidly.

Another point to consider is that we found that children with DCD made fewer saccades per trial, which could be argued to limit the conclusions if basic oculomotor function was interferring with social scene processing. Poor oculomotor control has in fact been reported in both ASD (Johnson et al. 2016) and DCD (Sumner et al. 2016a) populations when using paradigms designed to examine the execution of saccades and fixations. Such paradigms are notably different to a social scene task, as the latter in the present case does not impose any instructions for oculomotor behaviour (i.e., the present task involved passive viewing). Previous research using oculomotor paradigms suggests that children with DCD have difficulty inhibiting saccades and maintaining fixations when given specific instructions that test executive skills (Gonzales et al. 2016; Sumner et al. 2016a), however we find fewer saccades made during the social scene task than their peers (but not those with ASD) and no group differences in average fixation duration, which suggests that the children with DCD did not present with poor control of saccadic eye movements when participants are asked to freely view images. It was beyond the scope of this paper, but future research may investigate how fundamental oculomotor processes impact on spontaneous social processing for both children with ASD and DCD.

A final point to consider for future research relates to the gaze following analysis. The present study used images that contained only one object, whereas in other gaze following studies in adults with ASD the actor’s gaze is directed to one of two competing objects (the ‘congruent’ object; Freeth et al. 2010). Now that we have established potentially reduced tendency to follow gaze in DCD and ASD, future research using the congruent vs incongruent gaze approach in children ASD and DCD would be beneficial. It may be that individuals with ASD and DCD are able to use gaze information if they are directed to the eyes, but that this is not spontaneous. Regarding ASD, it has been suggested that this lack of automaticity of gaze following could be related to atypical functioning of the superior temporal sulcus, which is involved in the processing of dynamic aspects of faces (Pelphrey et al. 2005). Brain imaging studies in both ASD and DCD would be necessary to support this claim in relation to the current results.

In summary, the present study identified subtle differences between children with ASD and children with DCD. Those with ASD directed their attention to social aspects of a scene (the face, eyes) significantly less than their typically-developing peers, while children with DCD demonstrated less clear differences in their allocation of attention to these areas of interest compared to TD children. Exploratory analysis revealed that, in comparison to their TD peers, fewer children with DCD had a reduced tenedency to follow gaze in the social stimuli. Taken together, these findings hint at some atypicalities in allocating attention to social stimuli in DCD, but suggest that this may be linked to different causal mechanisms than in those with ASD. This work extends exisiting findings from standardised social measures to consider the allocation of visual attention to social stimuli. While this goes some way to improving our understanding of underlying processes affecting social behaviour in DCD, further research is required to understand the neural and cognitive underpinnings of social problems in the disorder. Future cross-syndrome comparisons are also warranted to aid understanding of the overlap of ASD and DCD and to determine effective avenues for intervention.

## Footnotes

1 The eye tracking analyses were also conducted excluding the 5 children with DCD that presented with high SCQ scores and the pattern of results remained the same. Therefore, the full sample is presented in subsequent analyses.

2 To further examine whether children with poor motor skill performed poorer than those with age-appropriate motor skills, the mean scores on the eye tracking measures for the subset of children with ASD performing below the motor cut-off on the MABC-2 were also compared to children with DCD and the remaining children with ASD who had age-appropriate motor skills. This analysis revealed no differences in the time spent fixating the face region (*p* = 0.09) or time to fixate the face (*p* = 0.17) between the three groups.
